# Predicting Skin-to-Bone Depth During Bone Marrow Biopsy: The Role of BMI, Waist Circumference, and Sex

**DOI:** 10.7759/cureus.115295

**Published:** 2026-08-27

**Authors:** A Niblock, Lisa Lyons, Kelly Ann Gillan, Kerrie Sweeney, Donal McLaughlin

**Affiliations:** 1 School of Medicine, Ulster University, Londonderry, GBR; 2 Haematology, Antrim Area Hospital, Antrim, GBR; 3 Laboratory Medicine, Antrim Area Hospital, Antrim, GBR

**Keywords:** body mass index (bmi), bone marrow biopsy, obesity, posterior superior iliac spine, waist circumfernce

## Abstract

Background

Bone marrow biopsy is an essential diagnostic procedure in haematology. Increasing rates of obesity may create technical challenges during biopsy by obscuring anatomical landmarks and increasing the distance between the skin surface and the posterior superior iliac spine (PSIS). Identifying patients who may be unsuitable for a standard non-image-guided approach could improve procedural planning, safety, and efficiency.

Methodology

Data from 50 consecutive patients undergoing bone marrow biopsy were collected as part of routine clinical care and a service evaluation. A retrospective analysis of these data was subsequently performed, with 46 patients included in the final analysis. Body mass index (BMI), waist circumference, and sex were recorded for each patient. Skin-to-bone depth at the PSIS was measured intra-procedurally. Associations were analysed using correlation analysis, simple linear regression, and multiple linear regression. Internal validation of the final regression model was performed using 1,000 bootstrap resamples to assess model stability and estimate optimism.

Results

Waist circumference and BMI were both significantly positively associated with skin-to-bone depth (p < 0.001 for both variables), with waist circumference demonstrating a stronger association (r = 0.617, R² = 0.381) than BMI (r = 0.489, R² = 0.239). Female patients demonstrated a greater mean skin-to-bone depth than males (4.74 cm vs 2.33 cm). A multiple linear regression model incorporating waist circumference and sex explained 61.1% of the variance in skin-to-bone depth (R² = 0.611). Internal validation using 1,000 bootstrap resamples demonstrated relatively stable model performance, with an optimism-corrected R² of 0.569. Although a waist circumference-by-sex interaction term was explored, it was not statistically significant.

Conclusions

Although waist circumference and sex were significant predictors of skin-to-bone depth, the predictive accuracy of anthropometric measurements alone was insufficient for reliable clinical decision-making. Substantial unexplained variability remained despite improved predictive performance in the combined regression model. Anthropometric measurements alone were therefore insufficient to reliably identify patients unsuitable for a standard non-image-guided biopsy. Clinical assessment remains essential when planning bone marrow biopsy procedures.

## Introduction

Bone marrow biopsy is a fundamental procedure in the diagnosis, staging, and monitoring of haematological disorders [1,2]. Traditionally performed by physicians, the procedure is increasingly undertaken by advanced practitioners, including nurses working in advanced practice roles, as multidisciplinary models of care continue to expand. Previous studies have demonstrated that appropriately trained practitioners can obtain diagnostic-quality samples safely and effectively, supporting increased procedural capacity and improved patient access [3-5]. However, identifying patients who are unsuitable for traditional non-image-guided procedures is essential to ensure appropriate triage to image-guided services and maintain clinic efficiency.

Historically, bone marrow biopsy has been regarded as a low-risk procedure, with rates of bleeding and infection reported to be less than 1% [6-8]. Anatomical landmarks can typically be identified through palpation, facilitating accurate localisation of the target site and appropriate guidance of the biopsy needle. Although any haematopoietic bones can potentially be biopsied, generally the posterior iliac crest at the point of the posterior superior iliac spine (PSIS) is considered the safest and most convenient site. It is large, readily accessible, close to the skin surface, outside the patient’s direct line of sight, and contains highly active marrow [9,10].

Increasing prevalence of obesity presents potential challenges for standard bone marrow biopsy. Excess adipose tissue may obscure anatomical landmarks, making localisation of the PSIS more difficult [11]. The excess tissue may also increase the distance between the skin surface and bone, leading to additional technical challenges and patient discomfort as the standard needles used for local anaesthetic administration or biopsy acquisition are insufficiently long. This may lead to failed initial biopsy attempts and necessitate repeat procedures, often under image guidance such as computed tomography (CT). Furthermore, increased tissue volume is associated with greater vascularity, which, combined with technical difficulty, has the potential to increase the risk of complications such as pain and bleeding.

Given the increasing rates of obesity and associated challenges, the ability to identify patients with increased skin-to-bone depth before biopsy may assist procedural planning and improve the experience of both patients and clinicians. Body mass index (BMI) and waist circumference are simple anthropometric measurements that can be easily collected in clinical practice, which may provide a practical means of estimating tissue depth. However, their ability to predict skin-to-bone depth at the PSIS has not been well characterised. Furthermore, recognised sex differences in body composition and fat distribution may influence tissue depth and procedural complexity. This study aimed to determine whether BMI, waist circumference and sex are associated with and can predict skin-to-bone depth at the PSIS, which was defined as the primary outcome measure. A secondary objective was to explore whether these anthropometric measurements might have potential utility in procedural planning for bone marrow biopsy.

## Materials and methods

This study represents a retrospective analysis of prospectively collected service evaluation data. Data were collected during routine clinical care between January 2025 and March 2025 within a nurse-led bone marrow biopsy service. Consecutive adult patients undergoing a standard non-image-guided PSIS bone marrow biopsy were considered eligible for inclusion. As this study represents a retrospective analysis of data collected during a service evaluation, no formal sample size calculation was performed before data collection. Consecutive eligible patients were included until data had been collected for 50 patients.

Height, weight, waist circumference and sex were recorded for each patient. BMI was calculated as weight (kg) divided by height squared (m²). To improve consistency between measurements, waist circumference was measured at the level of the superior border of the iliac crest in all patients.

Patients were eligible for inclusion in the analysis if they were aged 18 years or older, underwent a standard non-image-guided bone marrow biopsy at the PSIS within the nurse-led clinic, and had complete data available for all study variables, including BMI, waist circumference, sex, and skin-to-bone depth measurements. Patients undergoing image-guided bone marrow biopsy procedures were excluded, as were patients with incomplete datasets. Four patients were excluded because one or more study variables were missing, resulting in a final analytical sample of 46 patients.

During the procedure, skin-to-bone depth was measured directly. Once the biopsy needle had passed through the skin and subcutaneous tissues and contacted the cortical surface of the PSIS, a mark was made on the needle at the skin level. The distance from the needle tip to the skin mark was then measured and recorded as the skin-to-bone depth. Although this approach may be subject to minor variation arising from factors such as needle insertion angle and operator technique, it provided a simple and pragmatic method of obtaining direct intra-procedural measurements without reliance on imaging-based estimation. All patients were positioned in the lateral decubitus position with the upper knee flexed to facilitate biopsy at the PSIS. Procedures and measurements were undertaken by only two experienced nurses practising in advanced clinical roles within the nurse-led bone marrow biopsy service to minimise operator-related variability. Both practitioners had completed formal training and competency assessment in bone marrow aspiration and trephine biopsy procedures and had many years of experience performing these procedures independently. Needle selection was determined by the operator according to individual patient and procedural requirements, reflecting routine clinical practice. As measurements were collected as part of a service evaluation within routine clinical practice, formal inter-observer reliability assessment was not undertaken.

Associations between skin-to-bone depth and anthropometric measurements were initially assessed using Pearson correlation and simple linear regression analyses. BMI and waist circumference were evaluated individually as predictors of skin-to-bone depth. Sex-stratified analyses were subsequently performed to explore potential differences in these relationships between males and females. A multiple linear regression model incorporating waist circumference, sex, and a waist circumference-by-sex interaction term was then developed to evaluate the combined influence of these variables on skin-to-bone depth. This model was used to determine whether the relationship between waist circumference and skin-to-bone depth differed according to sex. For regression analyses, sex was coded as a binary variable (female = 0, male = 1). The interaction term was included to assess whether sex modified the relationship between waist circumference and skin-to-bone depth. Internal validation of the final regression model was performed using 1,000 bootstrap resamples to assess model stability and estimate optimism.

Statistical analyses were performed using Python 3.12 (Python Software Foundation) within Jupyter Notebook in Visual Studio Code. Data manipulation was performed using pandas 2.3.3, numerical computations using NumPy 2.3.5, statistical analyses using statsmodels 0.14.6, and data visualisation using Matplotlib 3.10.0 and multiple regression modelling with scikit-learn 1.9.0.

This project was conducted as a service evaluation using anonymised data collected as part of routine clinical practice. No alterations were made to patient care, and no identifiable patient information was collected for analysis. Formal Research Ethics Committee approval was therefore not required.

## Results

Data for 50 patients were collected, including sex, BMI, waist circumference, and skin-to-bone depth. Overall, four datasets were incomplete and were excluded from the analysis. Table 1 provides the data collected for each of the 46 patients (female n = 25, male n = 21).

**Table 1:** Recorded sex, BMI, waist circumference and skin-to-bone depth of all patients. BMI = body mass index; M = male; F = female

Patient	Sex	BMI (kg/m²)	Waist Circumference (cm)	Skin-to-bone depth (cm)
1	M	28.5	106	1
2	F	38.7	142	8
3	F	21.9	102	2.5
4	M	26	104	2
5	F	19.3	82	3
6	F	21	93	4
7	M	25	103	2
8	M	38.9	118	5
9	M	35	108	3
10	M	24.4	94	2
11	F	43	97	3
12	F	39	98	3
13	F	45	103	4
14	F	35	79	3
15	M	27.4	92	3
16	F	26.5	70	4
17	F	28.3	112	6
18	F	23.5	96	1
19	F	30.3	121	7
20	M	38.2	120	6
21	F	35.9	118	11
22	M	28.7	92	1
23	M	23	91	0.5
24	F	33	107	2
25	M	29	100	3
26	F	32	95	5
27	M	26.8	103	3
28	F	33.2	109	4
29	F	28.3	93	3
30	F	24.7	96	4
31	F	34.1	122	7
32	M	22.4	91	1
33	F	35.9	123	11
34	M	23	96	1
35	M	22.9	96	1
36	F	25.9	80	4
37	M	24.7	96	3
38	M	22.9	87	0.5
39	F	23	98	3
40	M	30	111	4
41	M	26	96	1
42	M	25	106	3
43	F	25	101	6
44	F	33.7	114	6
45	M	29.7	101	3
46	F	23	86	4

Sex

**Figure 1:**
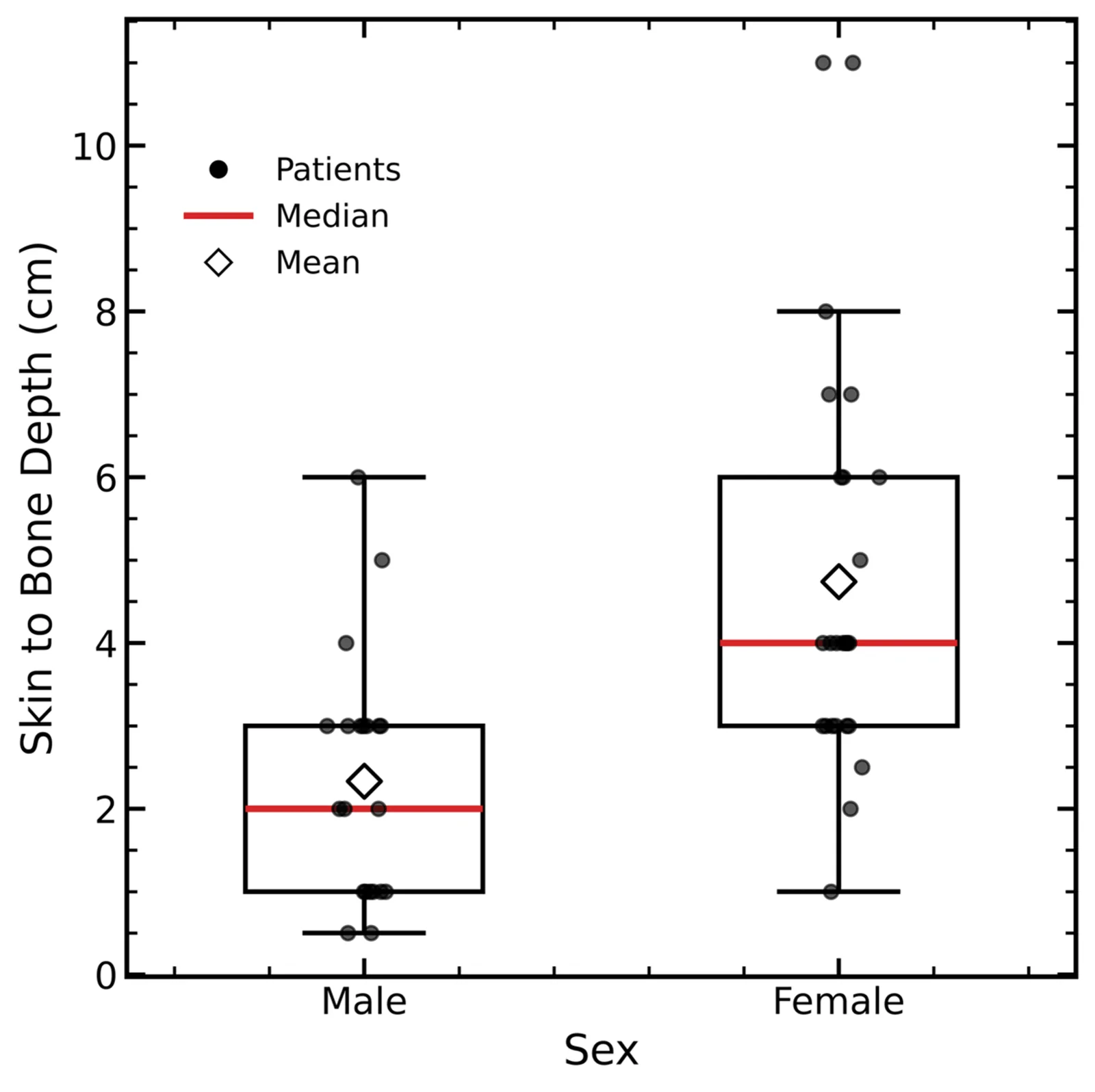
Effect of sex on skin-to-bone depth.

Skin-to-bone depth differed substantially between sexes (Figure 1). Based on the dataset, female patients had significantly greater skin-to-bone depth than male patients. Females exhibited a median depth of 4.0 cm (mean = 4.74 cm), compared with 2.0 cm (mean = 2.33 cm) in males. This difference was statistically significant (Mann-Whitney U = 97.0, p < 0.001)

Body mass index

**Figure 2:**
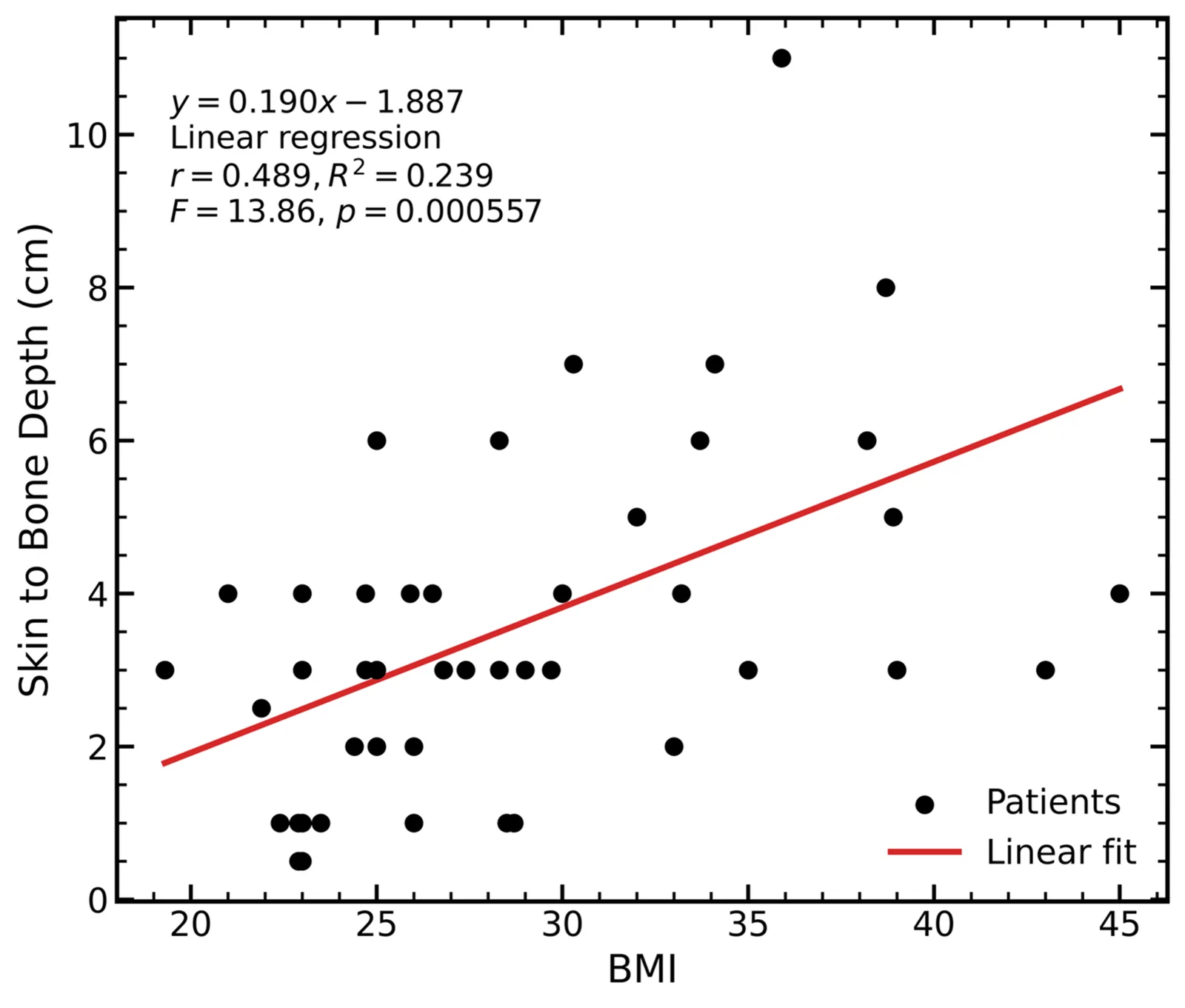
Scatter plot demonstrating the relationship of BMI with skin-to-bone depth. Simple linear regression demonstrated a significant positive association between BMI and skin-to-bone depth (r = 0.489, F = 13.86, p = 0.000557). BMI = body mass index

As shown in Figure 2, BMI demonstrated a statistically significant positive association with skin-to-bone depth. Linear regression showed that skin-to-bone depth increased by 0.19 cm for every 1 kg/m² increase in BMI (y = 0.190x − 1.887). The correlation was moderate (r = 0.489), with BMI explaining 23.9% of the variation in skin-to-bone depth (R² = 0.239). The regression model was statistically significant (F = 13.86, p = 0.000557), indicating a significant linear association between BMI and skin-to-bone depth. Although the overall trend was positive, substantial variable scatter around the fitted regression line was observed, particularly at higher BMI values, suggesting considerable inter-individual variability and indicating that factors other than BMI also contribute to skin-to-bone depth.

For example, two patients with an elevated BMI of 35.9 kg/m² demonstrated a skin-to-bone depth of 11 cm, substantially greater than most other participants with comparable BMI values. However, notably, several participants with relatively high BMI values demonstrated comparatively low skin-to-bone depths. For example, a participant with a BMI of 43 kg/m² had a skin-to-bone depth of only 3 cm, while patients with BMIs of 39 kg/m² and 45 kg/m² had depths of 3 cm and 4 cm, respectively.

Waist circumference

**Figure 3:**
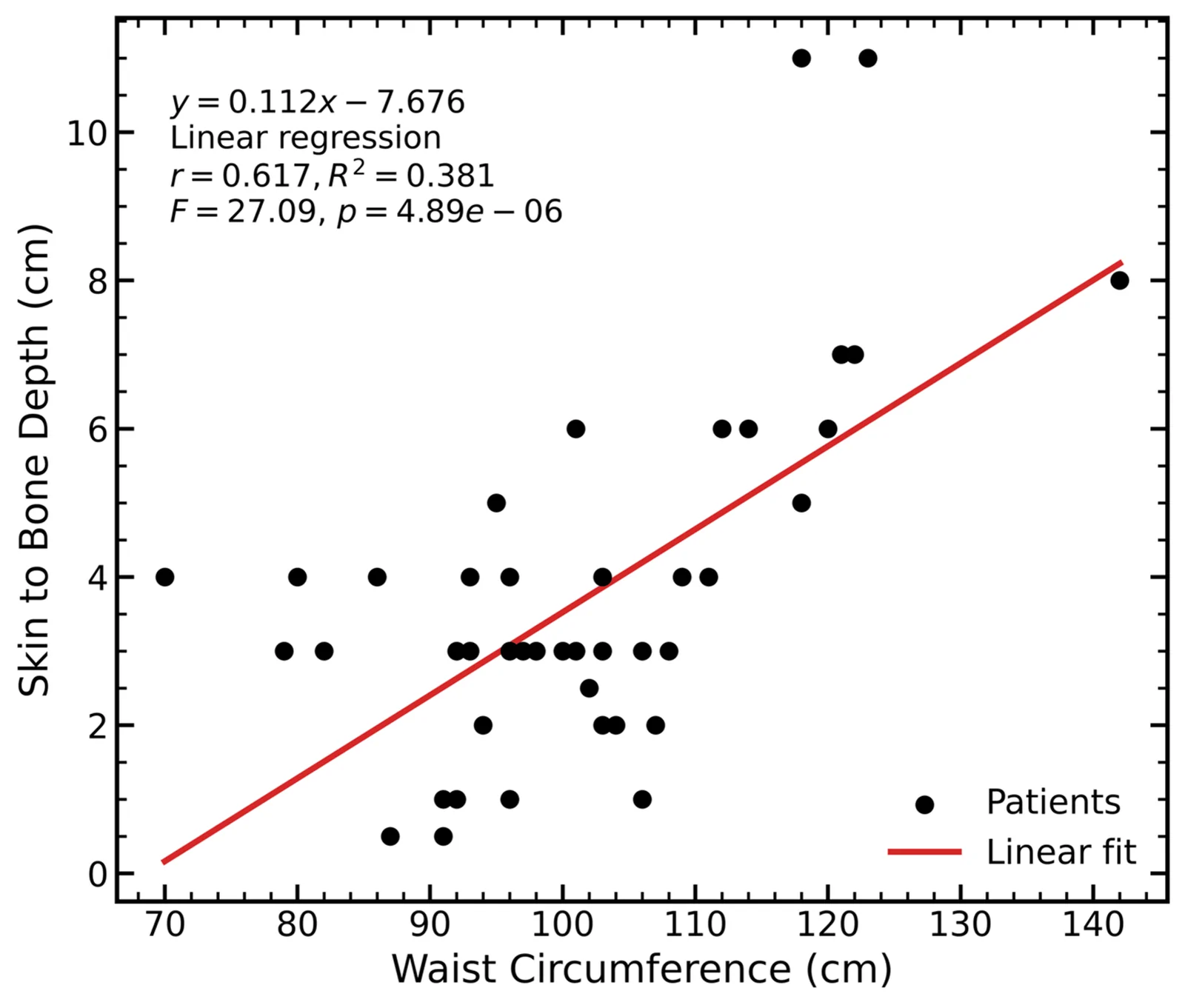
Scatter plot demonstrating the relationship of waist circumference with skin-to-bone depth. Simple linear regression demonstrated a significant positive association between waist circumference and skin-to-bone depth (r = 0.617, F = 27.09, p = 4.89 × 10⁻⁶).

Figure 3 demonstrates a significant positive association between waist circumference and skin-to-bone depth. Linear regression analysis showed that skin-to-bone depth increased by approximately 1 cm for every 10 cm (0.112 cm for every 1 cm) increase in waist circumference (y = 0.112x − 7.676). Pearson’s correlation indicated a moderately strong positive relationship (r = 0.617), with waist circumference accounting for 38.1% of the variance in skin-to-bone depth (R² = 0.381). The regression model was highly significant (F = 27.09, p = 4.89 × 10⁻⁶), indicating a significant linear association between waist circumference and skin-to-bone depth. Although the relationship was statistically significant, variability remained around the fitted regression line, indicating that additional patient characteristics also contribute to skin-to-bone depth.

Between waist circumference and BMI, waist circumference demonstrated a stronger relationship with skin-to-bone depth than BMI. Waist circumference exhibited a moderately strong positive correlation with skin-to-bone depth (r = 0.617, R² = 0.381, p = 4.89 × 10⁻⁶), explaining 38.1% of the observed variance. In comparison, BMI showed a more modest, although still statistically significant, association (r = 0.489, R² = 0.239, p = 5.57 × 10⁻⁴), accounting for 23.9% of the variance. The higher correlation coefficient, coefficient of determination, and F-statistic for waist circumference (F = 27.09 vs. 13.86) indicate that waist circumference is a more informative univariable predictor of skin-to-bone depth than BMI. However, neither variable explained more than 40% of the total variability, suggesting that additional factors influence skin-to-bone depth.

Differences between sex

**Figure 4:**
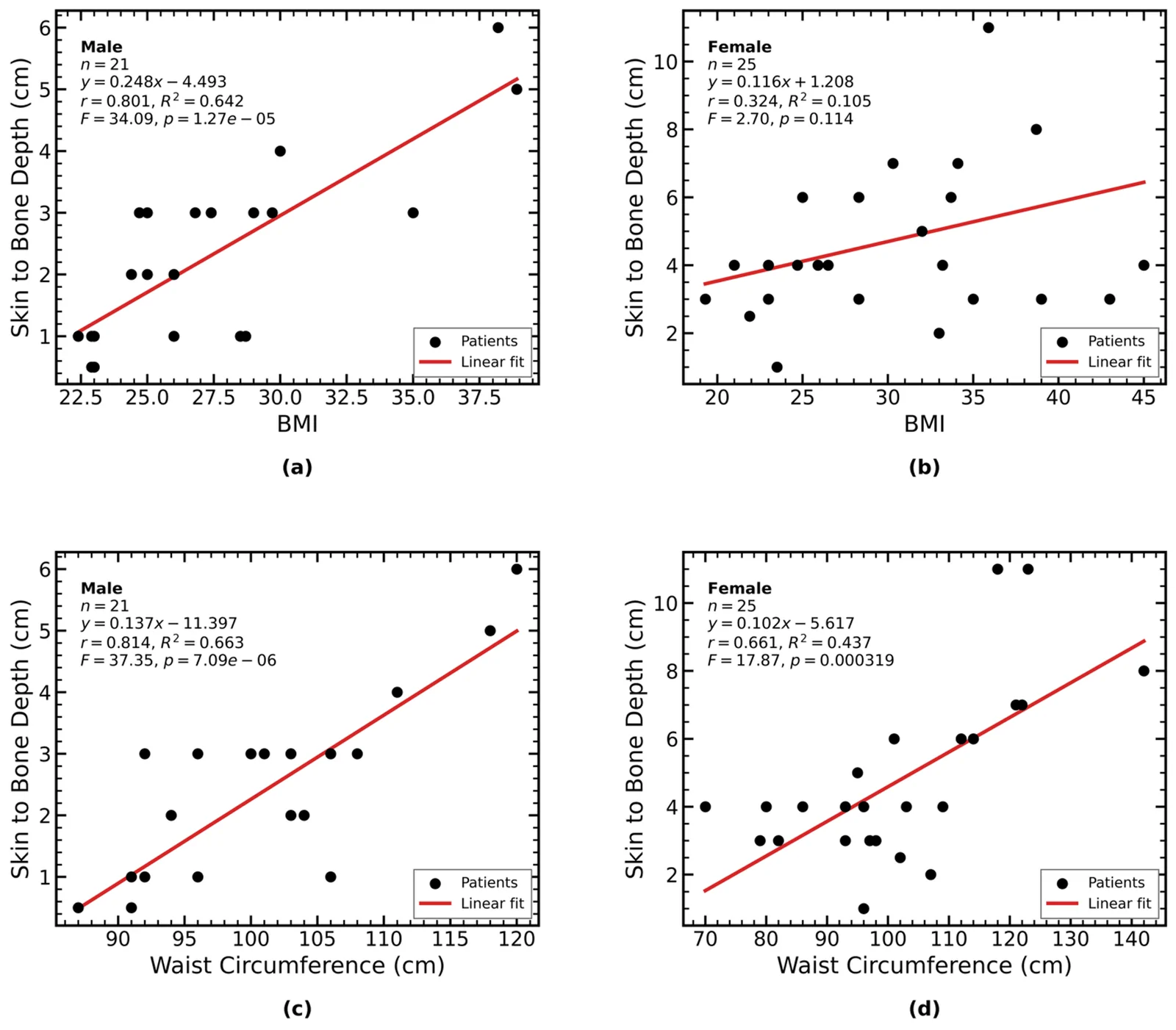
Relationships between skin-to-bone depth and anthropometric measures, bifurcated by sex. Panels (a) and (b) show BMI, while panels (c) and (d) show waist circumference. In males, BMI (r = 0.801, F = 34.09, p = 1.27 × 10⁻⁵) and waist circumference (r = 0.814, F = 37.35, p = 7.09 × 10⁻⁶) were significant predictors of skin-to-bone depth. In females, BMI was not significant (r = 0.324, F = 2.70, p = 0.114), whereas waist circumference remained a significant predictor (r = 0.661, F = 17.87, p = 3.19 × 10⁻⁴). BMI = body mass index

Sex-stratified analyses demonstrated marked differences in the relationship between anthropometric measures and skin-to-bone depth, as shown in Figure 4. In males (n = 21), BMI was strongly positively associated with skin-to-bone depth (r = 0.801, R² = 0.642, F = 34.09, p = 1.27 × 10⁻⁵), with skin-to-bone depth increasing by 0.248 cm per kg/m² increase in BMI. Waist circumference showed a similarly strong association in males (r = 0.814, R² = 0.663, F = 37.35, p = 7.09 × 10⁻⁶), explaining 66.3% of the variance in skin-to-bone depth. In females (n = 25), BMI was not significantly associated with skin-to-bone depth (r = 0.324, R² = 0.105, F = 2.70, p = 0.114). In contrast, waist circumference remained a significant predictor (r = 0.661, R² = 0.437, F = 17.87, p = 3.19 × 10⁻⁴), accounting for 43.7% of the observed variance. These findings indicate that waist circumference is a more consistent predictor of skin-to-bone depth than BMI across both sexes, whereas the predictive value of BMI appears to be largely confined to males.

Predictive model

Waist circumference was positively associated with skin-to-bone depth in both males and females. Sex-specific regression analyses showed a numerically stronger association in males (slope coefficient β = 0.137, R2 = 0.663) than in females (β = 0.102, R2 = 0.437); however, the waist circumference × sex interaction was not statistically significant (p = 0.433). Therefore, although the estimated slope was steeper in males, there was insufficient evidence to conclude that the relationship between waist circumference and skin-to-bone depth differed by sex.

Multiple linear regression incorporating waist circumference and sex was therefore used as the final predictive model. Sex was encoded as female = 0 and male = 1. Waist circumference was independently associated with skin-to-bone depth (β = 0.1089, 95% confidence interval (CI): 0.0740-0.1437, p < 0.001), corresponding to an estimated increase in skin-to-bone depth of approximately 1.09 cm for every 10 cm increase in waist circumference. Male sex was associated with a 2.30 cm lower predicted skin-to-bone depth compared with females at a given waist circumference (β = −2.3026, 95% CI: -3.2237 to -1.3815, p < 0.001).

The resultant additive predictive model was:

\begin{equation} \text{Skin to bone depth (cm)} = -6.31 + 0.1089 \times \text{waist circumference (cm)} - 2.30 \times \text{sex} \label{eq:placeholder_label} \end{equation}

The model explained 61.1% of the observed variation in skin-to-bone depth (R² = 0.611, adjusted R² = 0.593; F(2,43) = 33.77, p < 0.001). BMI was not included in the final regression model, with waist circumference and sex selected as the predictors in the final model. Internal validation using 1,000 bootstrap resamples demonstrated only a modest reduction in model performance, with an optimism-corrected R² of 0.569. Predictive error remained similar following validation, with a bootstrap root mean squared error of 1.55 cm and mean absolute error of 1.15 cm, compared with 1.49 cm and 1.10 cm, respectively, in the original model.

## Discussion

The findings highlight the limitations of commonly used anthropometric measurements in determining skin-to-bone depth in bone marrow biopsies. The variability observed across standard metrics reveals that BMI and waist circumference cannot act as standalone surrogates for internal tissue depth. While incorporating biological sex within a predictive model reduces some variability, the resulting explanatory power remains moderate (R² = 0.611) and is insufficient to support dependable clinical application. Because almost 40% of the variation in skin-to-bone depth remained unexplained, the model demonstrated insufficient predictive accuracy for routine clinical use. Although BMI and waist circumference showed statistically significant associations with tissue depth, the level of unexplained variability limits their usefulness for individual patient assessment. Nevertheless, internal bootstrap validation demonstrated only modest optimism, with the optimism-corrected R² decreasing from 0.611 to 0.569, suggesting that the observed model performance was relatively stable and not substantially driven by overfitting. Whilst skin-to-bone depth may contribute to procedural complexity, technical difficulty was not directly assessed in this study. Consequently, the findings should be interpreted as relating to variation in skin-to-bone depth rather than procedural success, failure, or biopsy difficulty.

An unexpected finding was the marked difference in predictive performance between sexes. In males, both BMI and waist circumference demonstrated strong associations with skin-to-bone depth, explaining over 60% of the observed variance. In contrast, BMI was not significantly associated with skin-to-bone depth in females. This may reflect recognised sex differences in body composition and fat distribution and suggests that the relationship between anthropometric measurements and tissue depth may not be uniform across patient groups.

The relatively poor performance of BMI is perhaps expected, given that it provides a measurement of overall body mass rather than local body composition. BMI is recognised as a useful population-level screening tool but does not directly measure adiposity, distinguish fat mass from lean mass, or account for regional fat distribution [12]. The moderate predictive performance observed in the present study suggests that skin-to-bone depth is likely influenced by factors beyond the anthropometric variables included in the model. The presence of both high-depth and low-depth outliers across a range of BMI values demonstrates substantial inter-individual variation in tissue depth. In particular, some patients with moderate BMI values exhibited unexpectedly large skin-to-bone depths, whereas others with very high BMI values had relatively low depths.

These findings support the conclusion that BMI alone is an unreliable predictor of skin-to-bone depth and that local anatomical factors are likely to contribute importantly to procedural variability. Previous research has demonstrated that soft-tissue thickness may vary according to age, sex, fat distribution patterns, and anatomical characteristics of the pelvis, indicating that local anatomical factors may influence tissue depth at the posterior iliac crest [13]. Obesity has been reported to make conventional landmark-guided bone marrow biopsy technically challenging because palpable anatomical landmarks may be difficult to identify. This difficulty frequently prompts patient referral for image-guided procedures [14,15].

Recent evidence demonstrates that patients referred for CT-guided bone marrow aspiration and biopsy have significantly higher BMIs and greater skin-to-bone depth than those undergoing bedside procedures, with large body habitus accounting for almost half of all referrals [16]. However, no significant differences in specimen quality have been observed between CT-guided and non-image-guided procedures, indicating that elevated BMI alone does not dictate procedural success or failure [16]. Furthermore, comparative data suggest that the relative performance of image-guided techniques improves primarily among patients with morbid obesity (BMI >40 kg/m²) [17]. This non-linear relationship implies that obesity contributes significantly to procedural complexity only at the extremes of body habitus, supporting the observation that broad anthropometric measures are insufficient to accurately predict tissue depth at the posterior iliac crest.

It is therefore likely that variation in individual skin-to-bone depth is driven by complex, individualised body composition factors rather than simple anthropometric measurements alone. The substantial variation in tissue depth observed between patients may be attributable to several factors. Biological differences, including individual and population-level variation in skeletal morphology and connective tissue composition, may contribute to differences in skin-to-bone depth independent of BMI [18]. Variability in muscle mass distribution, including muscular atrophy and hypertrophy, may also alter tissue depth in ways that are not reflected by anthropometric measurements alone [19]. Furthermore, the heterogeneous distribution of subcutaneous and visceral adipose tissue across different body types makes it difficult to assume a predictable or linear relationship between BMI and skin-to-bone depth [20]. Sex-specific differences in body composition and adipose tissue distribution may further contribute to variability in tissue depth, as women generally exhibit greater subcutaneous fat accumulation whereas men have relatively greater lean body mass and visceral adiposity [21].

These observations are further supported by the CT-guided biopsy literature, which repeatedly identifies difficulty locating anatomical landmarks in obese patients and suggests that skin-to-bone depth, rather than BMI itself, may represent the primary procedural challenge [14,15]. Patients referred for CT-guided procedures have been shown to possess substantially greater skin-to-bone depth than those undergoing bedside biopsy, reinforcing the concept that local anatomical characteristics may be more informative than conventional anthropometric measurements when assessing procedural difficulty [16].

From a practical perspective, these findings suggest that while increasing BMI or waist circumference may raise awareness of the possibility of greater skin-to-bone depth, neither measure should be used in isolation to determine biopsy approach. The marked variation in skin-to-bone depth among patients with similar anthropometric characteristics demonstrates that elevated BMI alone is not a reliable predictor of skin-to-bone depth. Consequently, clinical assessment and the ability to identify palpable anatomical landmarks may remain more useful for procedural planning than anthropometric measurements alone. Importantly, a high BMI should not automatically discourage attempts at bedside bone marrow biopsy where anatomical landmarks can be confidently identified.

As such, while further data collection may improve model performance incrementally, it is unlikely to substantially enhance predictive accuracy without the inclusion of additional variables beyond simple anthropometric measures. Given the limitations of anthropometric prediction demonstrated in this study, direct visualisation of local anatomy may offer a more reliable method of assessing skin-to-bone depth and local anatomical characteristics. In cases where uncertainty regarding skin-to-bone depth exists, consideration could be given to developing practitioner competency in pre-procedural or bedside ultrasound assessment. The role of ultrasound-guided biopsy techniques may also merit future investigation as a strategy to improve procedural accuracy and minimise technical challenges [22,23].

Several limitations should be acknowledged. The small sample size from a single centre may limit both the reproducibility and generalisability of the findings to broader patient populations. As this was an exploratory service evaluation, no formal sample size calculation was performed before data collection, and the findings should therefore be interpreted as hypothesis-generating. In addition, all procedures and measurements were performed by only two practitioners. While this approach was intended to minimise operator-related variability, it may limit the wider applicability of the findings and did not permit assessment of inter-observer reliability. Skin-to-bone depth was measured intra-procedurally rather than by imaging, introducing potential variability related to needle angle, tissue compression, and operator technique.

Furthermore, the predictive model was developed using a relatively small dataset of 46 participants. Internal validation using 1,000 bootstrap resamples was performed to assess model stability and estimate optimism. The optimism-corrected R² remained relatively stable at 0.569 compared with the original R² of 0.611, suggesting that model performance was not substantially driven by overfitting. However, external validation in an independent cohort was not undertaken. Consequently, the model should be considered preliminary and requires validation in larger, independent populations before its wider generalisability can be established.

The study also did not directly assess procedural outcomes such as biopsy failure, number of attempts, procedure duration, complications, patient discomfort, or the requirement for image-guided biopsy. Consequently, conclusions regarding procedural difficulty, biopsy success, procedural feasibility, or the need for image-guided biopsy cannot be drawn directly from these findings. Furthermore, the study was not designed to evaluate referral requirements for image-guided procedures, and no conclusions regarding the appropriateness of image-guided referral pathways can be drawn from the predictive model.

Finally, age and other potentially relevant patient characteristics, including body composition, muscle mass, and anatomical variation, were not assessed and may contribute to variation in skin-to-bone depth. Future studies involving larger multicentre cohorts and additional anatomical or imaging-based variables may help further characterise factors influencing tissue depth at the PSIS.

Future considerations may include piloting the use of bedside ultrasound to estimate skin-to-bone depth, as ultrasound has been shown to provide a reliable method for measuring subcutaneous tissue thickness and accurately identifying underlying anatomical structures. This approach has the potential to improve assessment of local anatomy and skin-to-bone depth before biopsy and may help identify patients who would benefit from further evaluation or alternative procedural approaches. However, its implementation would require additional training and upskilling of practitioners to ensure safe, effective, and consistent clinical application.

## Conclusions

From a practical perspective, these findings suggest that while waist circumference and female sex may serve as useful indicators of increased skin-to-bone depth and prompt further clinical assessment, they cannot be used in isolation to accurately predict skin-to-bone depth, determine procedural difficulty, or determine the need for image-guided biopsy. As procedural difficulty and biopsy success were not directly measured, conclusions regarding these outcomes cannot be drawn from the present study. Importantly, the absence of a clinically reliable predictive method reinforces the need for clinicians involved in bone marrow biopsies to rely on highly developed clinical judgement, procedural experience, and individualised patient assessment when determining the most appropriate biopsy approach. Practitioners should not necessarily be discouraged from attempting a bedside bone marrow biopsy solely based on a patient’s BMI. The substantial variation in skin-to-bone depth observed among individuals with similar anthropometric characteristics demonstrates that BMI alone is a poor predictor of skin-to-bone depth. Consequently, the ability to identify palpable anatomical landmarks may remain a more clinically meaningful indicator of likely procedural success than anthropometric measurements alone. This is particularly relevant within multidisciplinary services, where non-medical practitioners are required to balance autonomy with patient safety and recognise cases that may exceed the limitations of a non-image-guided technique. Surface-level anthropometric indices do not provide an accurate estimation of the depth to underlying bone targets; therefore, when standard biopsy is unsuccessful or technically challenging, liaison with medical colleagues and consideration of imaging modalities to ensure procedural safety and accuracy remain crucial, particularly in patients with increased adiposity.
